# Microbial contaminants isolated from items and work surfaces in the post- operative ward at Kawolo general hospital, Uganda

**DOI:** 10.1186/s12879-018-2980-5

**Published:** 2018-02-06

**Authors:** Ivan Sserwadda, Mathew Lukenge, Bashir Mwambi, Gerald Mboowa, Apollo Walusimbi, Farouk Segujja

**Affiliations:** 1 Department of Medical Microbiology, International Health Sciences University, P.O. Box 7782, Kampala, Uganda; 2 Department of Medical Microbiology, Immunology and Molecular Biology, School of Biomedical Sciences, College of Health Sciences, Makerere University, P.O Box 7072, Kampala, Uganda; 3 Department of Biomolecular Resources, College of Veterinary Medicine, Animal Resources and Biosecurity, Makerere University, P.O. Box 7062, Kampala, Uganda

**Keywords:** Nosocomial infections, Gram-positive, Gram-negative, Antimicrobial susceptibility

## Abstract

**Background:**

Nosocomial infections are a major setback in the healthcare delivery system especially in developing countries due to the limited resources. The roles played by medical care equipment and work surfaces in the transmission of such organisms have inevitably contributed to the elevated mortality, morbidity and antibiotic resistances.

**Methods:**

A total 138 samples were collected during the study from Kawolo general hospital. Swab samples were collected from various work surfaces and fomites which consisted of; beds, sink taps, infusion stands, switches, work tables and scissors. Cultures were done and the susceptibility patterns of the isolates were determined using Kirby Bauer disc diffusion method. Data was analyzed using Stata 13 and Microsoft Excel 2013 packages.

**Results:**

A total of 44.2% (61/138) of the collected swab specimens represented the overall bacterial contamination of the sampled articles. *Staphylococcus aureus* and *Klebsiella pneumoniae* accounted for the highest bacterial contaminants constituting of 75.4% (46/61) and 11.5% (7/61) respectively. Infusion stands and patient beds were found to have the highest bacterial contamination levels both constituting 19.67% (12/61). The highest degree of transmission of organisms to patients was found to be statistically significant for patient beds with OR: 20.1 and *P-value* 8X10^− 4^. Vancomycin, ceftriaxone and ciprofloxacin were the most effective antibiotics with 100%, 80% and 80% sensitivity patterns among the isolates respectively. Multi-drug resistant (MDR) *Staphylococcus aureus* accounted for 52% (24/46) with 4% (1/24) classified as a possible extensively drug resistant (XDR) whereas Gram negative isolates had 27% (4/15) MDR strains out of which 50%(2/4) were classified as possible pan-drug resistant (PDR).

**Conclusion:**

The high prevalence of bacterial contaminants in the hospital work environment is an indicator of poor or ineffective decontamination. The study findings reiterate the necessity to formulate drug usage policies and re-examine effectiveness of decontamination and sterilization practices within Kawolo general hospital. We also recommend installation of a sound Microbiology unit at the hospital to take on susceptibility testing to check on the empirical use of antibiotics as a way of reducing the rampant elevations in drug resistances.

## Background

Nosocomial infections have become increasingly an emerging threat to the health care system over the past decades. They have highly been attributed to poor disinfection, decontamination, sterilization of hospital articles as well as weak antimicrobial stewardship policies [1]. The post-operative ward in most cases harbors patients who have undergone intrusive surgical procedures like C-sections, hip replacements and tumor among others. These procedures render them highly vulnerable to sepsis from nosocomial pathogens.

Some of the consequences of this healthcare hurdle include prolonged hospital visits, increased antimicrobial resistance, and disabilities in the affected patients which greatly reduce the quality of life and productive human resource [2, 3]. As such, it is very crucial to understand the roles played by the hospital equipment and the environment in the prolonged maintenance of nosocomial infections and transmission. Whereas the hospital environment may serve as reservoirs for these organisms, their transmission to patients is mostly through hand contact [4–6].

Bacterial species have been estimated to cause the vast majority of these nosocomial infections followed by fungi and protozoa [7]. Several previous studies have ventured into isolation of nosocomial infection-causing organisms but the most often encountered species include organisms like *Staphylococcus aureus*, *Escherichia coli, Pseudomonas aeruginosa, Klebsiella species* [4, 8–10].

Emergence of multidrug-resistant organisms has further escalated this problem especially in resource deprived nations as a result of overuse, misuse and inadequate antimicrobial stewardship policies in the health management systems. The lack of hospital antimicrobial teams and strict adherence to treatment guidelines has led to the wide usage of broad spectrum and first line antibiotics thus the exacerbation of the resistance. Implications of this resistance are prolonged hospital stays and the gross economic burden incurred in treatment with consequently high morbidity and mortality rates [11–13]. Whereas this has been a major worldwide public health threat, there is very limited information about the extent of this problem in Uganda because of limited antimicrobial resistance surveillance. Therefore, this study aimed at establishing the nosocomial bacterial contaminants often found on hospital fomites, cover items and work surfaces, and their antibiotic susceptibility patterns predisposing patients to infection in the post- operative ward at Kawolo general hospital.

## Methods

### Study design and sampling technique

This was a cross sectional laboratory based study was carried out from June – December, 2015 at Kawolo General Hospital where a total of 138 swab specimens were collected from medical equipment which comprised of scissors, infusion stands and patient beds, and work surfaces which were composed of tables, sink taps and light switches.

Criterion followed for sample collection comprised of simple random sampling with consideration given to areas with constant hand contact. 23 swabs were collected for each mentioned article and work surface using a sterile swab moistened in normal saline from areas approximately 10 cm in diameter using standard operating procedures. The swab samples were placed tightly in well labeled swab caps and transported back immediately to the laboratory at International Health Sciences University and cultured.

### Ethics approval and consent to participate

This study was approved by the research and ethics committee of International Health Sciences University, Institute of Allied Health Sciences. Permission was also granted by the Kawolo general hospital administration and the International Health Sciences University laboratories where the experiments were performed from. All ethical laws and regulations governing scientific research were promptly adhered to during the study.

### Culture and isolation procedures

Upon arrival at the laboratory, the swabs were inoculated onto Blood agar and MacConkey culture media plates and incubated for 24 h at 37 °C and inspected for bacterial growth. The obtained colonies were subjected to Gram stain, hemolysis patterns on blood agar and colonial characteristics for their identification.

### Biochemical tests

Biochemical tests used in the identification and isolation of the microorganisms comprised of; catalase, coagulase, motility test, TSI, urease, indole, oxidase and Simon’s Citrate agar.

### Susceptibility tests

Disc diffusion method was used to assess the antibiotic susceptibility patterns of the isolates to a panel of selected antibiotics following guidelines prescribed by Clinical and Laboratory Standards Institute (CLSI 2014). All isolates were assessed using the same inocula which were standardized to 0.5 McFarland solution turbidity. The Gram-positive isolates obtained were tested against trimethoprim sulfamethoxazole (1.25/23.5 μg), penicillin (10 IU), erythromycin (15 μg), vancomycin (30 μg), cefoxtin (30 μg) and clindamycin (2 μg). Gram-negative bacterial isolates were tested with ciprofloxacin (5 μg), gentamicin (10 μg), trimethoprim sulfamethoxazole (1.25/23.5 μg), chloramphenicol (30 μg), nalidixic acid (15 μg) and ceftriaxone (30 μg).

*Staphylococcus aureus* (ATCC 25923), *Escherichia coli* (ATCC 25922) and *Pseudomonas aeruginosa* (ATCC 27853) were the two standard quality control strains incorporated in the study alongside the isolates.

## Results

**Fig. 1:**
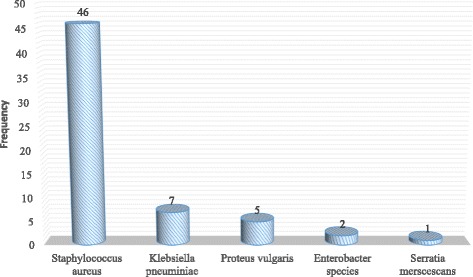
Frequency of the bacteria isolated from the swabs with bacterial growth

A total of 138 swabs were collected from medical care instruments and work surfaces from the post-surgical ward at Kawolo General Hospital. Among all the items sampled, 61 (44.2%) of them presented with bacterial growth. The isolates had 46 (75.4%) Gram-positive organisms all of which were *Staphylococcus aureus* while 15 (24.6%) were Gram-negatives that consisted of *Klebsiella pneumoniae* with 7(11.5%), *Proteus vulgaris, Enterobacter species* and *Serratia merscescans* representing 5(8.2%), 2(3.3%) and 1(1.6%) respectively (Fig. 1).

### Bacterial distribution profile on the swabbed items

Infusion stands and patient beds were the most likely to have the highest level of bacterial contaminants both accounting for 12 swabs (19.67%) followed by tables and sinks both represented by 10 (16.39%), scissors with 9 (14.75%) and light switches with 8 (13.11%) Distribution of bacterial contaminants in each item sampled in the study was tabulated and illustrated as shown in Table 1.

**Table 1:** Distribution of bacterial isolates on each respective medical item and work surface

Isolate	Infusion stands	Patient beds	Sink taps	Tables	Scissors	Light switches	Total
*Enterobacter spp*	1	0	0	0	0	1	2
*K. pneumoniae*	1	0	1	3	1	1	7
*Proteus vulgaris*	0	1	1	1	0	2	5
*S. merscescans*	1	0	0	0	0	0	1
*S. aureus*	9	11	8	6	8	4	46
Total (N)	12	12	10	10	9	8	
Percentage	19.67	19.67	16.39	16.39	14.75	13.11	

### Antibiogram profile for gram-positive isolates

**Fig. 2:**
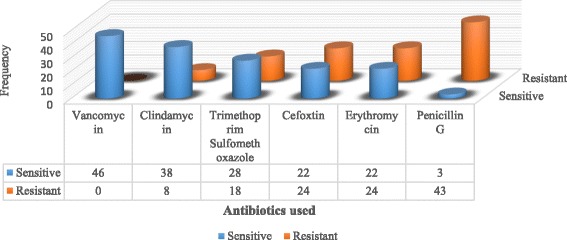
Gram-positive antimicrobial susceptibility pattern

*Staphylococcus aureus* which was the only Gram-positive bacterium isolated and was tested using an antibiotic panel consisting of: vancomycin, clindamycin, trimethoprim sulfamethoxazole, cefoxtin, erythromycin and penicillin G. The data for the susceptibility testing of *Staphylococcus aureus* is shown in fig. 2. Using cefoxtin as a surrogate marker, 24(52%) of *Staphylococcus aureus* isolates were defined as MRSA as they were resistant to Cefoxtin from which 1 isolate (4%) was defined as a possible XDR as per the guidelines provided by the CLSI 2014.

### Antibiogram profile for gram-negative isolates

**Fig. 3:**
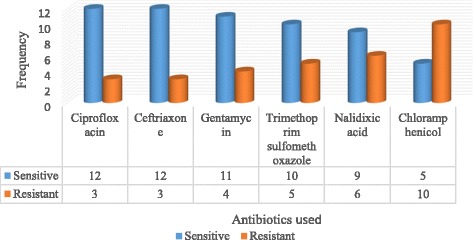
Gram-negative isolate antimicrobial susceptibility pattern

*Klebsiella pneumoniae* 7(47%), *Proteus vulgaris* 5(33%), *Enterobacter species* 2(13%) and *Serratia merscescans* 1(7%) were the isolates obtained in the constituting a total of 15 isolates. These were tested against a panel of antibiotics consisting of: ciprofloxacin, ceftriaxone, gentamycin, trimethoprim sulfamethoxazole, nalidixic acid and chloramphenicol. The data for the susceptibility testing of Gram negative isolates is shown in Fig. 3. The CLSI 2014 guidelines were used to further group the Gram-negative isolates according to their resistance degrees and the study revealed that 4(27%) out of the 15 isolates were classified as MDR. Out of the 4 MDR, *Klebsiella pneumoniae* had 1(25%) MDR and 2(50%) being further classified as possible PDR while *Proteus vulgaris* accounted for the remaining 1(25%) of the MDR strains. (Table 2).

**Table 2:** Classification of Gram-negative isolates according to their degree of resistance

Isolate	Frequency	Resistance degree
*Klebsiella pneumoniae*	2	PDR
*Klebsiella pneumoniae*	1	MDR
*Proteus vulgaris*	1	MDR

## Discussion

The overall prevalence of bacterial contamination in the post-operative surgical ward revealed by our study was 44% (61/138) which was considerably high and capable of giving rise to nosocomial infections. Such contamination is suggestive of a variety of factors which may include; poor decontamination, poor sanitation practices, ineffective disinfectants used and ineffective sterilization of medical care articles. Different prevalences have been reported in Uganda and elsewhere in similar studies. Jinja and Mulago hospitals in Uganda reported 58.5% and 68.8% respectively [9, 10] probably because the conditions in all these health facilities are somewhat similar. Elsewhere in Nigeria, a prevalence of 47% was reported [14] whereas 85.8% was reported from a research done at Jimma university teaching hospital in Ethiopia [15]. Beyond Africa, a research performed in Taiwan reflected a prevalence of 63.5% [16] and 57% from a study carried out in 7 hospitals in Iran [6]. The data from this study indicates the articles contaminated with bacteria have an increased risk as a source for nosocomial transmission of HAIs.

*Staphylococcus aureus* with 75.4% (46/61) was the most prevalent bacterial contaminant isolated by our study. These results coincide with those from other studies where *Staphylococcus aureus* had the highest prevalence of 30.2% in a study done in Nigeria [4] and 28.4% in Cameroon [1]. *Staphylococcus aureus* is naturally occurring as a normal flora on human mucosa. The presence of surface proteins present in their cell walls for biofilm, evasion of host immune responses and tissue adhering abilities has enabled these organisms to survive and flourish in nature [17].

*Klebsiella pneumoniae* 11.5% (7/15), was the most predominant Gram-negative we isolated in our study. Isolation of such enterobacteria is highly suggestive of fecal contamination, poor personal hygiene and most especial poor hand washing practices amongst health workers and patients. The high prevalence of this organism is attributed to the presence of pili encoded by *mrkD* gene in *Klebsiella pneumoniae* that aid in adherence of the organism to host tissues during invasion and biofilm bacterial forms are 10 times more resistant to antibiotics than the uncapsulated forms [18].

Patient beds and infusion stands had the highest bacterial contamination levels of 19% (12/61) compared to the other surfaces and equipment swabbed. This is because these are some of the articles most frequently in contact with hands during patient care in any hospital setting as most authors have reported those medical workers’ hands are the most probable means of transfer [19]. In order to assess the effectiveness of the decontamination protocols used in the hospital, the research team consulted the hospital administration and found out that ordinary liquid soap and sodium hypochlorite were used for cleaning the hospital twice a day utmost which was basically done for the hospital floors. On top of that, the sterilizer for ensuring sterility of medical equipment before use was not regularly serviced. Other articles like infusion stands, work tables and beds were seldom decontaminated and yet they were found to harbor infectious organisms. There were also tales of cleaning agents occasionally running out of stock. We deduced that the persistence of the bacteria in the hospital environment could be attributed to the ineffective strength of disinfecting agents and limited frequency of decontamination. The beds are responsible for accommodating a vast number of the patients that are admitted within the post-operative ward and yet rarely or no sterilization is done to ensure their decontamination. This renders them highly exposed and most likely responsible for harboring such organisms that can later result into nosocomial infections.

Antimicrobial resistance poses one of the greatest threats to the survival of mankind. Indeed, the antimicrobial resistance review (2015) highlighted an associated global mortality of closely 700,000 deaths per annum with a projection of approximately 10 million deaths by 2050. As for the current study, vancomycin was the most effective antibiotic against Staphylococcal infections as demonstrated by our study. As such, it empirically recommended in treatment of suspected MRSA cases; however, reports of increasing vancomycin-resistant *Staphylococcus aureus* have been on the rise over the years. Increased resistance levels were observed for penicillin, cefoxtin and erythromycin with 93%, 48% and 48% resistances respectively. This could be attributed to the increasing overuse and misuse of antibiotics by the general population as a result of a weak health management system since they are readily available over the counter even without clinicians’ prescriptions. These same reasons can be used to explain the high frequency of MRSA that represented 52% (24/46) out of which 4% (1/24) was described as a possible XDR.

Ciprofloxacin, ceftriaxone and gentamycin with 80%, 80% and 73% sensitivities respectively were the most effective antibiotics as per our study. On the contrary, chloramphenicol and nalidixic acid with 67% and 40% resistances respectively had the least sensitivities. Increasing resistances are reflective of ready availability and prescription abuse. MDR enterobacteria accounted for 27% (4/15) out of which *Klebsiella pneumoniae* had 1(25%) possible PDR resistant to all the antibiotics in the drug profile. Our study findings on MDR organisms therefore coincide with many similar studies [1, 20] and call for immediate intervention to slow down this public health threat.

Among our shortcomings, our study had a small sample size which gave a small reflection about the magnitude of the challenge and we therefore recommend a nationwide similar study to get a bigger picture of this problem. Also antimicrobial susceptibility patterns were determined using phenotypic disc diffusion methods; however, we recommend whole genome sequencing for deciphering the resistome of these bacteria.

## Conclusion

Our study, being the first of its kind to be done at Kawolo general hospital found out that *Staphylococcus aureus* and *Klebsiella pneumoniae* were the most predominant isolates most likely to cause HAIs. Hospital fomites have been found to harbor disease-causing microbes and are easily transmitted through hand contact from one person to another. Mutations and other various factors have aided these organisms to mount resistance towards commonly used antibiotics which has rendered treatment of infectious diseases inefficient.

There is indeed a dire need to scale up and strengthen the existing infection control strategies at Kawolo hospital, and the entire country through reassessment of the strengths and types of the decontaminants used to clean hospitals, frequency of decontamination or sterilization, good personal hygiene, and establishment of a national surveillance program to monitor and control HAIs.

## Abbreviations

%: Percent
°C: Degree Celsius
ATCC: American Type Culture Collection
CLSI: Clinical Laboratory Standards Institute
cm: Centimeter
HAI: Hospital Acquired Infection
MDR: Multi Drug Resistant
MRSA: Methicillin Resistant *Staphylococcus aureus*
OR: Odds Ratio
PDR: Pan Drug Resistant
TSI: Triple Sugar Iron
XDR: Extensive Drug Resistance
